# Gender differences in the efficacy of pioglitazone treatment in nonalcoholic fatty liver disease patients with abnormal glucose metabolism

**DOI:** 10.1186/s13293-020-00344-1

**Published:** 2021-01-04

**Authors:** Hongmei Yan, Weiyun Wu, Xinxia Chang, Mingfeng Xia, Sicheng Ma, Liu Wang, Jian Gao

**Affiliations:** 1 Department of Endocrinology and Metabolism, Zhongshan Hospital, Fudan University, Shanghai, 200032 China; 2 Fudan Institute for Metabolic Disease, Fudan University, Shanghai, 200032 China; 3 Department of Laboratory, Zhongshan Hospital, Fudan University, Shanghai, 200032 China; 4 Shanghai Starriver Bilingual School, Shanghai, 201108 China; 5 Second Affiliated Hospital of Army Military Medical University, Chongqing, 400037 China; 6 Department of Nutrition, Zhongshan Hospital, Fudan University, Shanghai, 200032 China

**Keywords:** Pioglitazone, Gender, Liver fat content, Nonalcoholic fatty liver disease, Abnormal glucose metabolism

## Abstract

**Background:**

Pioglitazone is a promising therapeutic method for nonalcoholic fatty liver disease (NAFLD) patients with or without type 2 diabetes. However, there is remarkable variability in treatment response. We analyzed our previous randomized controlled trial to examine the effects of gender and other factors on the efficacy of pioglitazone in treating Chinese nonalcoholic fatty liver disease (NAFLD) patients with abnormal glucose metabolism.

**Methods:**

This is a post hoc analysis of a previous randomized, parallel controlled, open-label clinical trial (RCT) with an original purpose of evaluating the efficacy of berberine and pioglitazone on NAFLD. The total population (*n* = 185) was randomly divided into three groups: lifestyle intervention (LSI), LSI + pioglitazone (PGZ) 15 mg qd, and LSI + berberine (BBR) 0.5 g tid, respectively, for 16 weeks. The study used proton magnetic resonance spectroscopy (^1^H-MRS) to assess liver fat content.

**Results:**

As compared with LSI, PGZ + LSI treatment further decreased liver fat content in women (− 15.24% ± 14.54% vs. − 8.76% ± 13.49%, *p* = 0.025), but less decreased liver fat content in men (− 9.95% ± 15.18% vs. − 12.64% ± 17.78%, *p* = 0.046). There was a significant interaction between gender and efficacy of pioglitazone before and after adjustment for age, smoking, drinking, baseline BMI, BMI change, treatment adherence, baseline liver fat content, and glucose metabolism.

**Conclusion:**

The study recommends pioglitazone plus lifestyle intervention for Chinese NAFLD female patients with abnormal glucose metabolism.

**Trial registration:**

Role of Pioglitazone and Berberine in Treatment of Non-Alcoholic Fatty Liver Disease, NCT00633282. Registered on 3 March 2008, https://register.clinicaltrials.gov.

## Introduction

Nonalcoholic fatty liver disease (NAFLD) is a condition defined by the presence of steatosis in more than 5% of hepatocytes with little or no alcohol consumption [1]. It comprehends a spectrum of diseases that spans from simple hepatic steatosis to nonalcoholic steatohepatitis (NASH), defined histologically by the presence of hepatic steatosis with evidence for hepatocellular ballooning, lobular inflammation, and almost always fibrosis [1]. As NAFLD is strongly associated with all the components of metabolic syndrome, some experts suggested that “metabolic associated fatty liver disease (MAFLD)” is a more appropriate term than the acronym NAFLD [2]. The global incidence of NAFLD is increasing rapidly and will probably emerge as the leading cause of chronic liver disease among patients with obesity, prediabetes, or type 2 diabetes (T2DM) [3]. NAFLD patients with T2DM are considered to be at a higher risk of developing progressive liver diseases, as well as extra-hepatic complications [4]. Currently, the treatment of NAFLD is mainly lifestyle interventions, and there is no recognized drug with expected efficacy for clinical use. Most of the therapeutic drugs in phase 2b and phase 3 clinical trials barely meet the anticipated liver histological endpoint. Some studies indicate that pioglitazone, vitamin E, liraglutide, and obeticholic acid might be promising drugs for NAFLD [5, 6]. However, vitamin E and obeticholic acid have some adverse effects such as lipid metabolism disorders, skin itching, and their safety of long-term use have not been confirmed [7, 8]. As to liraglutide, its clinical application is limited for potential risks of pancreatitis and medullary thyroid cancer [6]. Pioglitazone, being used to treat T2DM, has been under scrutiny for associated adverse effects such as heart failure and fracture risk [9, 10]. However, it shows a promising therapeutic prospect in NAFLD/NASH medication intervention [11].

Thiazolidinediones (TZDs), as a class of glucose-lowering agents, mediate their actions through the activation of peroxisome proliferator-activated receptor gamma (PPAR-γ) to improve insulin sensitivity [12]. PPAR-γ is a transcription factor found in adipocytes, macrophages, monocytes, hepatocytes, muscle, and endothelial cells, and it controls the expression of genes involved in glucose and fatty acid metabolism, energy storage, and inflammatory response [13–15]. Pioglitazone, belonging to TZDs, is a potent PPAR-γ and a less dominant PPAR alpha (PPAR-α) agonist [16, 17]. PPAR-α plays a pivotal role in the modulation of hepatic lipid metabolism, oxidative stress, inflammatory response, and fibrogenesis [18]. As reported by previous studies, pioglitazone was associated with significant histologic improvement in terms of steatosis, inflammation, NAFLD activity score, resolution of NASH, and fibrosis in Western NASH patients with or without T2DM [19, 20]. Favorable effects have also been shown in Asian populations [21, 22]. Our previous research showed a significant decrease in liver fat content (LFC) with pioglitazone treatment in NAFLD patients with abnormal glucose metabolism [23]. Based on current evidence, the 2017 American Association for the Study of Liver Diseases (AASLD) guidelines proposed that pioglitazone could be used to treat biopsy-proven NASH patients [1]. However, the histological improvement in the liver caused by pioglitazone did not happen in all patients. For example, only 47% of the patients achieved the primary outcome in the PIVENS (Pioglitazone versus Vitamin E versus Placebo for the Treatment of Nondiabetic Patients with Nonalcoholic Steatohepatitis) trial [19]. Therefore, it is necessary to identify those individuals who are likely to respond best to certain treatment options in order to increase benefits of a given intervention.

Some studies have investigated the factors affecting the efficacy of pioglitazone, most of which are related to the effects on reducing blood glucose, and few are related to the treatment of fatty liver. Factors involved in the various response to pioglitazone in patients with T2DM were gender, BMI, baseline levels of fasting plasm glucose (FPG), and circulating levels of endorphin [24, 25]. Female and obese patients with higher FPG levels, higher BMI, or higher concentration of endorphin are more likely to show a significant reduction in glycated hemoglobin (HbA1c) after pioglitazone treatment [24, 25]. As to the efficacy of NALFD/NASH treatment, several studies identified that pioglitazone exposure index and adiponectin levels may account for the response variability [26, 27]. Since these two parameters are not easy to acquire or measure, they are not the optimal indicators used to judge the efficacy of drugs before selecting medication treatment. We are trying to find a simple and effective clinical parameter, such as gender, BMI, and so on, to prejudge drug therapeutic effect before choosing medication. Herein, in this post hoc analysis, we would like to assess whether gender could be an appropriate indicator to identify efficacy of pioglitazone in Chinese NAFLD patients with abnormal glucose metabolism.

## Materials and methods

### Study design

The data come from a total of 185 NAFLD patients with impaired glucose regulation (IGR) or T2DM, who participated in a clinical trial (NCT00633282) in the Department of Endocrinology, Zhongshan Hospital, Fudan University, from 2008 to 2012, which is a randomized, parallel controlled, open-label clinical trial with three-arm. A detailed description of the RCT has previously been published [23]. Briefly, participants were divided into three groups: lifestyle intervention (LSI), LSI + pioglitazone (PGZ) 15 mg qd, and LSI + berberine (BBR) 0.5 g tid. The treatment lasted for 16 weeks. Safety-related events, adherence, pill counts, and serum samples were collected. Inclusion criteria include age 18–70 years, fatty liver diagnosed by ultrasound, and individuals with prediabetes (impaired glucose regulation, IGR) or T2DM: fasting plasm glucose ≥ 5.6 mmol/L and/or 2 h postprandial glucose ≥ 7.8 mmol/L. Exclusion criteria were those who have already used hypoglycemic drugs, those with poor glycemic control (HbA1c ≥ 9.5%), and those who are pregnant, breastfeeding, or have severe illness. LSI was conducted following the standardized recommendation (500 kcal calorie less per day than before and 150 min medium-intensity or 90 min high-intensity aerobic exercise per week) [28]. The Ethics Committee of Zhongshan Hospital, Fudan University, approved this trial.

### Measurement of liver fat content using ^1^H-MRS

LFCs were detected by proton magnetic resonance spectroscopy (^1^H-MRS) using a 1.5-T magnetic resonance (MR) scanner (Siemens Avanto, Erlangen, Germany) equipped for proton spectroscopy acquisitions. Sagittal, coronal, and axial slices covering the whole liver were preliminarily acquired for positioning of the spectroscopy acquisition voxel. Signal intensities of water peak at 4.8 ppm (Sw) and the fat peak at 1.4 ppm (Sf) were measured and hepatic fat percentage was calculated using the formula 100 × Sf/(Sf + Sw). The details have been described in the published study [23].

### Statistical methods

R software 3.4.3 was used for statistical analysis. Kolmogorov-Smirnov test was carried out to determine the normality of the continuous variables. Quantitative data was expressed as mean ± SD if it was normally distributed, or median and interquartile range if it was not. Categorical variables were expressed as frequency (or percentage). Differences between before and after intervention were tested by paired *t* test or Wilcoxon rank test. Differences between males and females were tested by unpaired Student’s *t* test or Mann-Whitney *U* test for quantitative variables and the *χ*^2^ test or Fisher’s exact test for qualitative variables. Differences in LFC changes between groups were expressed as mean difference (95% confidence interval). Interaction tests were included in the linear regression model as the product of gender and study grouping and were assessed by Wald’s test. *p* < 0.05 was defined as a statistically significant difference.

## Results

### Basic characteristics and intervention results among men and women

Of 185 participants, 155 have completed the study, including 85 male individuals and 70 female individuals. Female patients were older and had lower rate of smoking and drinking than male patients (all *p* < 0.001). There were no differences about adherence, baseline BMI (28.0 ± 3.5 vs. 27.4 ± 4.0), final BMI (27.0 ± 3.1 vs. 26.5 ± 4.1), baseline LFC (%) (33.02 ± 13.70 vs. 36.01 ± 16.21), final LFC (%) (19.65 ± 12.65 vs. 21.76 ± 15.79), and absolute changes of LFC (%) (− 13.35 ± 15.41 vs. − 14.27 ± 15.58) between male and female patients (all *p* > 0.05) (Table 1). Absolute changes of LFC (%) are the absolute changes separately in three groups or three groups combined. No significant differences in baseline fasting plasm glucose (FPG) (6.28 ± 1.27 mmol/L vs. 6.22 ± 1.09 mmol/L, *p* = 0.532) or 2 h postprandial glucose (2hPG) (11.59 ± 3.77 mmol/L vs. 11.41 ± 3.76 mmol/L, *p* = 0.875) were observed between male and female patients (Supplemental Table 1).

**Table 1:** Basic characteristics and intervention results among men and women

	Men (*n* = 85)	Women (*n* = 70)	*P* value
Group, *n* (%)			0.220
LSI	28 (32.94%)	25 (35.71%)	
PGZ + LSI	22 (25.88%)	25 (35.71%)	
BBR + LSI	35 (41.18%)	20 (28.57%)	
Age (age)	49.62 ± 10.81	53.47 ± 7.68	0.013
Smoking, *n* (%)	23 (27.06%)	2 (2.86%)	< 0.001
Drinking, *n* (%)	25 (29.41%)	1 (1.43%)	< 0.001
Glucose metabolism			0.643
IGR, *n* (%)	42 (49.41%)	37 (52.86%)	
T2DM, *n* (%)	43 (50.59%)	33 (47.14%)	
Treatment adherence (%)	100 (15–100)	100 (32–100)	0.161
Baseline BMI (kg/m^2^)	28.0 ± 3.5	27.4 ± 4.0	0.301
Final BMI (kg/m^2^)	27.0 ± 3.1	26.5 ± 4.1	0.466
BMI changes (kg/m^2^)	− 1.1 ± 1.1^a^	− 0.9 ± 1.1^a^	0.270
Baseline LFC (%)	33.02 ± 13.70	36.01 ± 16.21	0.215
Final LFC (%)	19.65 ± 12.65	21.76 ± 15.79	0.357
Absolute changes of LFC (%)	− 13.35 ± 15.41^a^	− 14.27 ± 15.58^a^	0.714
LSI (*n* = 53)	− 12.64 ± 17.78^a^	− 8.76 ± 13.49^a^	0.379
PGZ + LSI (*n* = 47)	− 9.95 ± 15.18^a^	− 15.24 ± 14.54^a^	0.229
BBR + LSI (*n* = 55)	− 16.06 ± 13.35^a^	− 19.95 ± 17.59^a^	0.359

Data are given as means ± SD or median (Min-Max) for continuous variables and percentages for categorical variables. Absolute changes of LFC (%), the absolute changes separately in three groups or three groups combined. *P* value calculated by unpaired Student’s *t* test or Mann-Whitney *U* test for quantitative variables, and χ^2^ test or Fisher’s exact test for qualitative variables, differences between women and men

*IGR* impaired glucose regulation, *T2DM* type 2 diabetes, *BMI* body mass index, *LSI* lifestyle intervention, *PGZ* pioglitazone, *BBR* berberine, *LFC* liver fat content

^a^Calculated by paired *t* test, differences between before and after intervention

### Effects of gender on changes in liver fat content after treatment

Changes of LFC stratified by genders are shown in Table 2.

**Table 2:** Interaction between changes of LFC and gender among three groups

	No. of patients	Absolute changes of LFC (%) β (95%CI)	*P* value*	Interaction test *P* value
PGZ + LSI vs LSI				
Men	50	9.79 (0.37, 19.21)	0.046	0.003
Women	50	− 8.26 (− 17.18, − 0.65)	0.025	
BBR + LSI vs LSI				
Men	63	− 1.50 (− 9.38, 6.38)	0.710	0.124
Women	45	− 11.88 (− 21.61, − 2.14)	0.020	
BBR + LSI vs PGZ + LSI				
Men	57	− 11.29 (− 18.99, − 3.58)	0.007	0.222
Women	45	− 3.61 (− 13.61, 6.38)	0.483	

Data are shown as means (95% confidence interval). Absolute changes of LFC (%), the difference of the absolute changes of LFC for men or women between the PGZ + LSI and LSI groups, the BBR + LSI and LSI groups, or the BBR + LSI and PGZ + LSI groups. Interaction test *P* value was assessed by Wald’s test

*LFC* liver fat content

*P value calculated by linear regression model, difference between two different treatment groups.

After treatment, the absolute value of LFC was decreased by 11.4% and 12.1%, respectively, in the LSI group and the PGZ + LSI group [23]. It is worth noting that, relative to group LSI, the LFC of group PGZ + LSI was further decreased in female patients [− 8.26% (− 17.18%, − 0.65%), *p* = 0.025], whereas, it was less decreased in male patients [9.79% (0.37%, 19.21%), *p* = 0.046]. A significant interaction between gender and pioglitazone’s efficacy was observed (*p* = 0.003). Compared with LSI, BBR + LSI treatment caused further decreased LFC in female patients [− 11.88% (− 21.61%, − 2.14%), *p* = 0.020], while it did not cause significant changes in male patients [1.50% (− 9.38%, 6.38%, *p* = 0.710]. No interaction between gender and efficacy was found in the BBR + LSI group (*p* = 0.124). Compared with PGZ + LSI, BBR + LSI intervention was associated with significant reduction of LFC in males [− 11.29% (− 18.99%, − 3.58%), *p* = 0.007], and no significant changes in females [− 3.61% (− 13.61%, 6.38%), *p* = 0.483]. No interaction between gender and efficacy was found between PGZ + LSI and BBR + LSI intervention (*p* = 0.222).

### Interaction between changes of LFC and gender in the PGZ + LSI group compared with the LSI group in different models

After adjustment for age, smoking, drinking, baseline BMI, BMI changes, and treatment adherence, the interaction test between gender and efficacy remained significant (model 2, *p* = 0.011). After adjusted for baseline LFC and variables in model 2, gender difference was still significant (model 3, *p* = 0.039). Gender difference was also significant after adjusted for glucose metabolism and variables in model 2 (model 4, *p* = 0.024). However, gender difference was not significant after adjusted for the change of HOMA-IR and variables in model 2 (model 5, *p* = 0.059).

## Discussion

To the best of our knowledge, this is the first study to validate that gender is an independent factor affecting the pioglitazone’s efficacy on LFC in Chinese NALFD patients with abnormal glucose metabolism. Thus, based on lifestyle intervention, the study shows that prescribing pioglitazone further reduces LFC in women. However, the same effect is not significant for the male counterpart. The sex-specific difference between the PGZ + LSI and LSI groups could partially result from the fact that LSI alone induced greater decrease in LFC in men. This study suggests that in populations with NALFD and abnormal glucose metabolism, women may profit more from adding pioglitazone treatment to lifestyle intervention than men.

Studies have shown that gender differences existed in the effects of TZDs on patients with diabetes or obesity. TZDs were more effective in women than in men in glycemic control and lipids improvement. Clinical Practice Research Datalink (CPRD) found that male patients and lower BMI were associated with a poorer response with TZDs (both *p* < 0.001) [29]. A Diabetes Outcome Progression Trial (ADOPT) and Rosiglitazone Evaluated for Cardiovascular Outcomes and Regulation of Glycemia in Diabetes (RECORD) reported that obese females had a greater HbA1c reduction with TZDs than that with sulfonylureas (*p* < 0.001) [29]. Additionally, triglycerides decreased significantly in women but not in men with pioglitazone treatment (*p* = 0.015) [30]. Our previous study showed that after pioglitazone treatment, women experienced a greater drop in blood glucose and insulin (Additional file 1: Supplemental Tables 1 and 2. On contrary, for overweight or obese individuals, pioglitazone intervention combined with energy-restricted diet and resistance training for 16 weeks, abdominal visceral fat was significantly reduced in men rather than in women [31]. However, few researches have reported the gender differences of pioglitazone on NAFLD. The PIVENS study did subgroup analysis and revealed no sex difference in the effects of pioglitazone in patients with NASH [19]. But in this study, we found that pioglitazone has gender differences in the treatment of NAFLD patients with abnormal glucose metabolism, and pioglitazone is more favorable for female patients. The differences between these two studies might be due to the different clinical characteristics of subjects or racial differences. Future clinical trials should focus more on sex differences in drug efficacy in different races and ethnicities.

In this study, after pioglitazone treatment, homeostasis model assessment of insulin resistance (HOMA-IR) tended to decrease more in women than in men (Additional file 1: Supplemental Table 1). The interaction between changes of LFC and gender in the PGZ + LSI group compared with the LSI group became insignificant after adjusted for the changes of HOMA-IR (Table 3). Thus, gender disparity of pioglitazone response on LFC may be related to the gender disparity of pioglitazone on insulin resistance. However, there is little research on the sex-based difference of pioglitazone effect on insulin resistance, which could be explored in future studies.

**Table 3:** Interaction between changes of LFC and gender in the PGZ + LSI group compared with the LSI group in different models

	Absolute changes of LFC (%)	Men	Women	Interaction test *P* value
Model 1	*β* (95%CI)	9.79 (0.37, 19.21)	− 8.26 (− 17.18, − 0.65)	0.003
	*P* value*	0.046	0.025	
Model 2	*β* (95%CI)	8.42 (− 1.40, 18.23)	− 8.19 (− 16.64, − 0.27)	0.011
	*P* value*	0.099	0.033	
Model 3	*β* (95%CI)	5.25 (− 3.25, 18.76)	− 7.75 (− 12.32, − 0.13)	0.039
	*P* value*	0.464	0.045	
Model 4	*β* (95%CI)	6.09 (− 3.02, 15.20)	− 5.25 (− 13.38, 2.88)	0.024
	*P* value*	0.193	0.210	
Model 5	*β* (95%CI)	5.86 (− 3.24, 14.97)	− 4.78 (− 13.43, 3.88)	0.059
	*P* value*	0.211	0.283	
Model 6	*β* (95%CI)	6.98 (− 6.59, 14.54)	− 3.33 (− 11.09, 4.44)	0.291
	*P* value*	0.801	0.405	

Data are shown as means (95% confidence interval). Absolute changes of LFC (%), difference of the absolute changes of LFC for men or women between the PGZ + LSI and LSI groups. Model 1: not adjusted; model 2: adjusted for age, smoking, drinking, baseline BMI, change of BMI, and treatment adherence; model 3: adjusted for baseline LFC and variables in model 2; model 4: adjusted for glucose metabolism and variables in model 2; model 5: adjusted for change of HOMA-IR and variables in model 2; model 6: adjusted for baseline LFC, glucose metabolism, change of HOMA-IR, and variables in model 2. Interaction test *P* value was assessed by Wald’s test

*LFC* liver fat content

*P value calculated by linear regression model, difference between the PGZ + LSI group and the LSI group

It is generally believed that the difference of circulating sex hormone level is one of the main factors underlying the gender differences. Androgen, one of the sex hormones, has inconsistent effects on fatty liver between men and women. For example, reduced testosterone levels in men were associated with an increased risk of NAFLD [32], whereas in women, elevated circulating testosterone levels increased the prevalence of NAFLD [33]. In addition, testosterone deficiency increases visceral fat content and insulin resistance in men, while in women, high androgen levels increase insulin resistance and visceral fat [34]. As a classical agent with sex-disparity effects, pioglitazone has been reported to both decrease testosterone levels in men with diabetes [35] and in women with polycystic ovary syndrome (PCOS) [36]. Thus, the sex-based difference in fatty liver observed in our study might be owing to the paradox influence of androgen levels between different sexes. Estrogen, another sex hormone, has shown protective actions in NAFLD/NASH [37]. Although the effects of TZDs on estrogen levels have not been reported in humans, estrogen may influence PPAR-γ expression and function in an animal study, showing that 17β-estradiol significantly upregulated PPAR-γ protein expression in a concentration-dependent manner [38]. Taken together, gender disparity in response of pioglitazone is likely due to differences in sex hormones, especially the level of androgen. Therefore, the effect of pioglitazone on sex hormones deserves further studies.

Gender differences in pioglitazone action might be also related to the different pharmacological effects of pioglitazone between the two sexes. The clearance rate of pioglitazone in female mice was slower than that in male mice. After single oral administration or continuous oral administration, the blood active metabolic concentrations in female mice were higher than that in male mice [39]. CYP2C8 is a critical enzyme in the metabolism of pioglitazone, and the CYP2C8 genotype could be a potential factor for the sex difference. Carriers of the CYP2C8*3 allele have faster metabolism rate of pioglitazone and have less improvement in liver fibrosis after pioglitazone intervention (*p* = 0.026) [40]. However, it has not been reported whether there are gender differences in the expression of CYP2C8 in humans, and only one study found that the mRNA and protein levels of CYP2C8 in the liver of white individuals were independent of gender [41]. Hence, there is a need for further studies to verify whether the pharmacokinetic of pioglitazone participates in the gender differences in LFC.

A substantial body of evidence has demonstrated that pioglitazone exerts salutary effects on metabolic syndrome, improves NAFLD/NASH, and reduces cardiovascular events; there are still concerns about the adverse effects especially the increased rate of congestive heart failure reported in PROactive (The Prospective Pioglitazone Clinical Trial in Macrovascular Events) trial [10]. Therefore, pioglitazone prescription should be avoided in patients with higher risk of heart failure. However, the potential risks do not appear to negate the beneficial effects and these side effects can be mitigated by optimizing dosing strategies and combining therapy with other medications in appropriate patients [11].

From another perspective, according to Tables 1 and 2, LSI alone tended to induce greater numerical reduction of LFC in male than that in female (− 12.64% ± 17.78% vs. − 8.76% ± 13.49%, *p* > 0.05 ), while PGZ + LSI tended to induce less reduction of LFC in male than that in female (− 9.95% ± 15.18% vs. − 15.24% ± 14.54%, *p* > 0.05). It means that sex-specific difference between the PGZ + LSI and LSI groups might partially result from the fact that LSI alone induced greater decrease in LFC in males. Few earlier studies have shown that men respond better to lifestyle intervention than women. In the study of Torgerson and co-workers, a very-low-calorie diet program was a successful treatment for some severely obese subjects, especially men [42]. In line with this result, Pekkarinen et al. found that men were more likely to acquire a 5-year maintenance of weight loss after very-low-energy diets together with behavior therapy [43]. Consistently, in a Weight Control for Life-program, men lost more weight and maintained better losses than women [44]. However, the reasons and mechanisms why men respond better to lifestyle interventions than women are not further explained in these articles.

This study has several limitations. Firstly, as this is a post hoc analysis, residual confounding cannot be eliminated. The results of this study are only used as the basis for hypothesis generation and more well-designed clinical trials with large population should be conducted to clarify this finding further. Secondly, this study did not detect the hepatic histological lesion and whether there are gender differences in liver histology remains unknown. Further trials using liver histology as the main observation outcome are necessary to evaluate the gender-specific differences in fatty liver of pioglitazone therapy. Thirdly, the sample size is relatively small, and larger-scale clinical trials should be conducted to confirm this provocative finding.

## Conclusions

In this study, gender differences were found in Chinese NAFLD patients with abnormal glucose metabolism treated with pioglitazone. Female patients, rather than male patients, were more appropriate to be prescribed pioglitazone plus lifestyle intervention.

## Perspectives and significance

There are no therapies currently licensed for the treatment of nonalcoholic fatty liver disease (NAFLD). Pioglitazone may be used to treat biopsy-proven NASH patients with or without T2DM due to its ability to improve liver histology. However, in the PIVENS trial (pioglitazone, vitamin E, placebo for non-diabetic, and nonalcoholic steatohepatitis), less than half of the patients achieved histological improvements in the liver using pioglitazone. In this study, we found that gender was one of the main factors affecting the efficacy of pioglitazone in Chinese NAFLD patients with glucose metabolism disorder. When pioglitazone was added to lifestyle intervention, women with NAFLD profited more than men. These results remind us of paying more attention to the influence of gender biology on the response to disease treatment. This study also suggests that, when selecting an optimal therapeutic strategy for NAFLD in clinical practice, factors affecting the efficacy of drugs should be more carefully considered to tailor individualized treatment for patients.

## Supplementary Information

**Additional file 1: Supplemental Table 1**. Characteristics before and after treatment in PGZ+LSI group. **Supplemental Table 2**. Interaction between changes of LFC and gender among three groups.

## Data Availability

All the data generated or analyzed during this study are included in this published article.
