# Supplementary material for: Gender differences in the efficacy of pioglitazone treatment in nonalcoholic fatty liver disease patients with abnormal glucose metabolism
Source: Biol Sex Differ. 2021 Jan 4;12:1. doi: 10.1186/s13293-020-00344-1 (PMC7784274; doi:10.1186/s13293-020-00344-1)
Supplement: Supplementary file 1 — Additional file 1: Supplemental Table 1. Characteristics before and after treatment in PGZ+LSI group. Supplemental Table 2. Interaction between changes of LFC and gender among three groups. [file 13293_2020_344_MOESM1_ESM.docx]

**Supplemental data**

Supplemental Table 1 Characteristics before and after treatment in PGZ + LSI group

|  | Pre-treatment | | | | Post-treatment | | |
| --- | --- | --- | --- | --- | --- | --- | --- |
|  | Men | Women | | *P* value | Men | Women | *P* value |
| FPG | 6.28 ± 1.27 | 6.22 ± 1.09 | | 0.532 | 6.49 ± 1.03 | 5.69 ± 0.79 | 0.062 |
| 2hPG | 11.59 ± 3.77 | 11.41 ± 3.76 | | 0.875 | 9.21 ± 3.14 | 8.21 ± 2.41 | 0.223 |
| HbA1c (%) | 6.49 ± 0.64 | | 5.97 ± 0.56 | 0.731 | 6.56 ± 0.73 | 6.00 ± 0.46 | 0.812 |
| FINS | 15.18 ± 8.29 | | 14.75 ± 6.86 | 0.832 | 15.68 ± 7.87 | 12.34 ± 6.10 | 0.210 |
| HOMA-IR | 4.35 ± 2.84 | | 4.06 ± 1.90 | 0.872 | 4.47 ± 2.34 | 3.09 ± 1.54 | 0.062 |
| LFC (%) | 29.55 ± 14.70 | | 19.68 ± 13.78 | 0.045 | 39.28 ± 17.32 | 24.00 ± 21.37 | 0.422 |

Data are given as Means ± SD; FPG, fasting plasm glucose; 2hPG, 2h post prandial glucose; HbA1c, glycated hemoglobin; FINS, fasting serum

insulin; HOMA-IR, homeostasis model assessment of insulin resistance; LFC, liver fat content. *P* value calculated by unpaired Student’s *t* test,

differences between women and men.

Supplemental Table 2 Interaction between changes of LFC and gender among three groups

|  |  | PGZ+LSI vs LSI | | BBR+LSI vs LSI | | BBR+LSI vs PGZ+LSI | |
| --- | --- | --- | --- | --- | --- | --- | --- |
|  |  | Men | Women | Men | Women | Men | Women |
| Model 1 | Changes of LFC (%) β (95%CI) | 9.79 (0.37, 19.21) | -8.26 (-17.18, -0.65) | -1.50 (-9.38, 6.38) | -11.88 (-21.61, -2.14) | -11.29 (-18.99, -3.58) | -3.61 (-13.61, 6.38) |
|  | **P* value | 0.046 | 0.025 | 0.710 | 0.020 | 0.007 | 0.483 |
|  | Interaction *P* value | 0.003 | | 0.124 | | 0.222 | |
|  |  |  |  |  |  |  |  |
| Model 2 | Changes of LFC (%) β (95%CI) | 5.25 (-3.25, 8.76) | -7.75 (-12.32, -0.13) | -3.67 (-10.18, 2.83) | -11.89 (-20.56, -3.23) | -4.59 (-10.84, 1.66) | -8.09 (-17.09, 0.91) |
|  | **P* value | 0.464 | 0.045 | 0.272 | 0.040 | 0.156 | 0.086 |
|  | Interaction *P* value | 0.039 | | 0.009 | | 0.802 | |
|  |  |  |  |  |  |  |  |
| Model 3 | Changes of LFC (%) β (95%CI) | 6.09 (-3.02, 15.20) | -5.25 (-13.38, 2.88) | -1.82 (-9.88, 6.24) | -13.80 (-23.14, -4.47) | -6.95 (-14.56, 0.67) | -8.50 (-18.11, 1.10) |
|  | **P* value | 0.193 | 0.210 | 0.658 | 0.005 | 0.079 | 0.091 |
|  | Interaction *P* value | 0.024 | | 0.182 | | 0.691 | |
|  |  |  |  |  |  |  |  |
| Model 4 | Changes of LFC (%) β (95%CI) | 5.86 (-3.24, 14.97) | -4.78 (-13.43, 3.88) | -1.34 (-9.33, 6.66) | -16.41 (-26.00, -6.82) | -5.95 (-13.40, 1.51) | -10.20 (-20.99, 0.60) |
|  | **P* value | 0.211 | 0.283 | 0.744 | 0.001 | 0.124 | 0.072 |
|  | Interaction *P* value | 0.059 | | 0.134 | | 0.902 | |

Data are showed as Means (95% confidence interval); LFC, liver fat content; Model 1: not adjusted; Model 2: adjusted for age, smoking, drinking, baseline BMI, change of BMI, and treatment adherence; Model 3: adjusted for baseline LFC and variables in Model 2; Model 4: adjusted for glucose metabolism and variables in Model 2; Model 5: adjusted for change of HOMA-IR and variables in Model 2; Model 6: adjusted for baseline LFC, glucose metabolism, change of HOMA-IR, and variables in Model 2. β, mean difference between the two treatment groups; *P value calculated by linear regression model, difference between the two treatment groups; Interaction test P value was assessed by Wald test.
